# The relationship between probiotics and retinopathy of prematurity in preterm infants: A population-based retrospective study in China.

**DOI:** 10.3389/fped.2023.1055992

**Published:** 2023-02-21

**Authors:** Wen Hua Bai, Dan Feng Gu, Yun Dai, Yu Hong Chen, Zu Ming Yang, Li Jun Lu

**Affiliations:** Department of Neonatology, Suzhou Municipal Hospital, Suzhou, China

**Keywords:** probiotics, retinopathy of prematurity, preterm infants, bronchopulmonary dysplasia, severe intraventricular hemorrhage

## Abstract

**Background:**

Retinopathy of prematurity (ROP) is a retinal vascular disease with a high incidence in premature infants and is a leading cause of childhood blindness worldwide. The purpose of our study was to analyze the association between the use of probiotics and retinopathy of prematurity.

**Methods:**

This study retrospectively collected clinical data of premature infants with gestational age <32 weeks and birth weight <1500 g admitted to the neonatal intensive care unit from January 1, 2019 to December 31, 2021 in Suzhou Municipal Hospital, China. Demographic and clinical data of the inclusion population were collected. The outcome was the occurrence of ROP. The chi-square test was used to compare categorical variables, while the t-test and the nonparametric Mann-Whitney U rank-sum test were used for continuous variables. Univariate and multivariate logistic regression were used to analyze the relationship between probiotics and ROP.

**Results:**

A total of 443 preterm infants met the inclusion criteria, of which 264 didn't receive probiotics and 179 were supplemented with probiotics. There were 121 newborns with ROP in the included population. The results of univariate analysis showed that the preterm infants with and without probiotics were significantly different in the gestational age, the birth weight, the one-minute Apgar score, the oxygen inhalation time, the acceptance rate of invasive mechanical ventilation, the prevalence of bronchopulmonary dysplasia, ROP and severe intraventricular hemorrhage and periventricular leukomalacia (*P* < 0.05). Unadjusted univariate logistic regression model result showed that probiotics (OR 0.383, 95% CI 0.240∼0.611) were the factors affecting ROP in preterm infants (*P* < 0.01). Multivariate logistic regression result (OR 0.575, 95% CI 0.333∼0.994) was consistent with univariate analysis (*P* < 0.05).

**Conclusion:**

This study showed that probiotic was associated with a reduced risk of ROP in preterm infants with gestational age of <32 weeks and birth weight of <1500 g, but more large-scale prospective studies are still needed.

## Introduction

Retinopathy of prematurity (ROP) is a retinal vascular proliferative disease that occurs in premature and low birth weight infants (1), and it is an important cause of blindness and severe visual impairment in preterm infants (2). Along with the increase of the premature infants' survival around the world, the incidence of ROP has also increased. The incidence of ROP varies greatly among different countries, which is mainly related to the level of economy and medical care (3). In China, the incidence of ROP in preterm infants ranges from 11.9% to 15.2% (4). A multi-center study on ROP in 7 administrative regions of China in 2015 showed that the incidence of ROP in preterm infants with birth weight less than 1000 g was 60% (5), while it was 42.4% in the United States from 2010 to 2015 (6).

There are many risk factors affecting the occurrence of ROP. It is well known that low gestational age, low birth weight and prolonged oxygen use are major risk factors of ROP. Other factors that may affect the occurrence of ROP include hyperglycemia, blood transfusion, asphyxia, intraventricular hemorrhage, sepsis, perinatal infection and inflammation (7–11).

The pathogenesis of ROP is mainly divided into two stages. The first stage is blockage and arrest of retinal blood vessels development. The second stage is secondary neovascularization caused by retinal hypoxia (12). A variety of factors have been found to be involved in neovascularization, such as vascular endothelial growth factor, insulin-like growth factor-1, epidermal growth factor, etc. At present, the treatment of ROP mainly includes laser therapy, anti-vascular endothelial growth factor drug therapy, scleral cerclage and vitrectomy (13). There are many pathogenic factors of ROP, and the pathogenesis is very complex. There is no single preventive mechanism, and comprehensive preventive measures should be taken.

Recent meta-analyses have shown that probiotic supplementation can shorten the time to reach full enteral feeding and reduce the risk of neonatal necrotizing enterocolitis (NEC) and late-onset sepsis (LOS) in preterm infants (14, 15), but there are few studies on whether probiotics affect the occurrence of ROP. Some studies have shown that the establishment of early gut microbiota can prevent the occurrence of severe ROP (16), but other meta-analyses have shown that the use of probiotics in preterm infants does not affect the risk of ROP (17). The purpose of this study is to investigate the association between probiotic use and ROP in preterm infants.

## Methods

We retrospectively collected the clinical data of newborns admitted to the neonatal intensive care unit of Suzhou Municipal Hospital from January 1st, 2019 to December 31st, 2021.

### Study population

**Figure 1:**
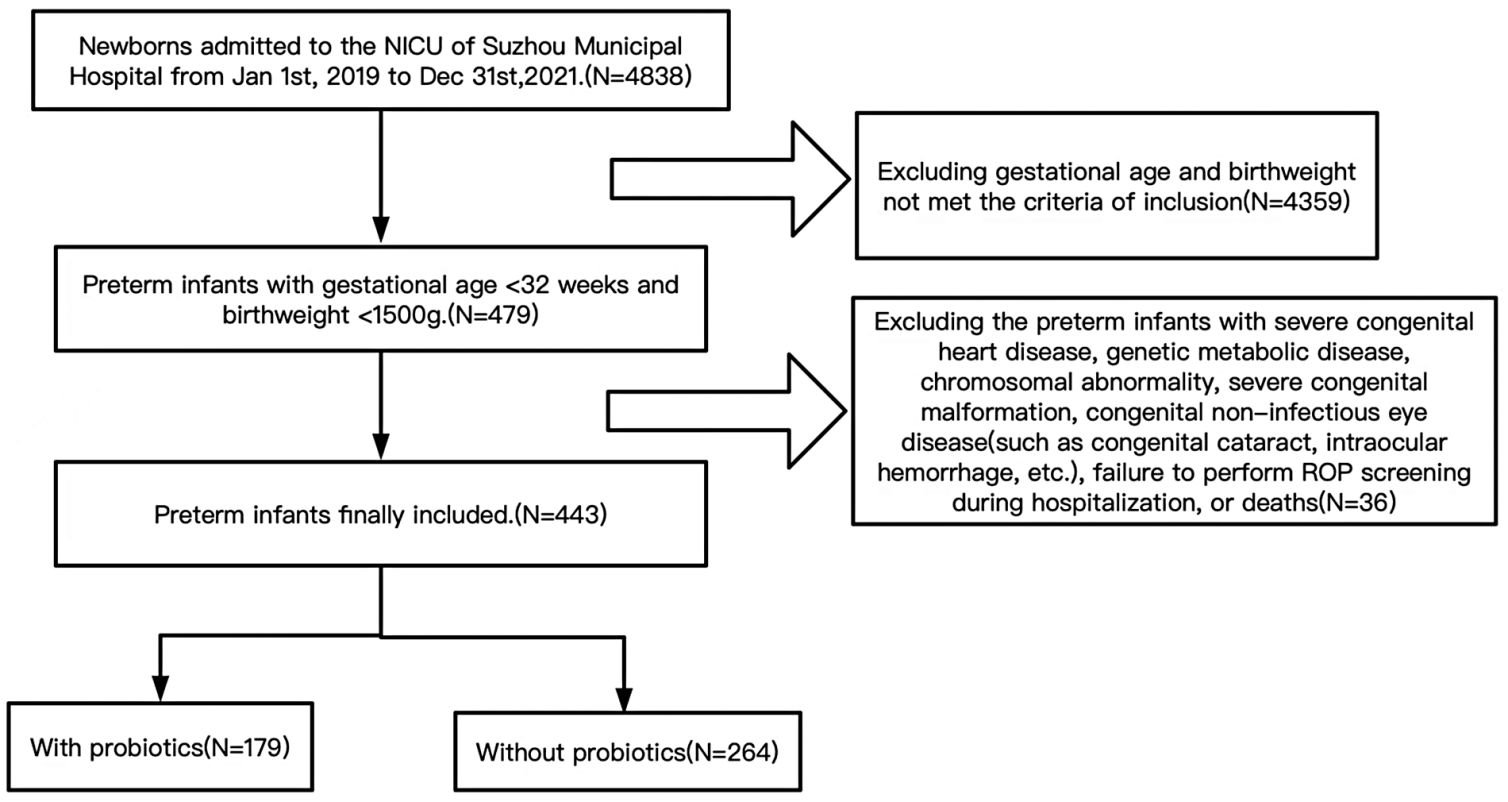
Flowchart of study population selection.

Preterm infants admitted to the neonatal intensive care unit within 24 h after birth with gestational age <32 weeks and birthweight <1500 g were included in the study. Preterm infants with severe congenital heart disease, genetic metabolic disease, chromosomal abnormality, severe congenital malformation, congenital non-infectious eye disease (such as congenital cataract, intraocular hemorrhage, etc.), failure to perform ROP screening during hospitalization, or deaths were excluded. Please see the flowchart in Figure 1.

### Study exposure factors and covariates

The main study subject in this study was probiotics. The probiotics used in our neonatal intensive care unit were Live Bifidobacterium Capsules [Lizhu Pharmaceutical Group Co., Ltd, 0.35 g/capsule, each capsule containing 5 × 10^9^ colony forming units, one tablet each time, twice a day]. Currently, probiotics are mainly used clinically for feeding intolerance and pathological jaundice (18, 19). In this study, probiotics group were defined as those who took oral probiotics lasting for ≥ 7 days within three weeks after birth.

Covariates included demographic and clinicopathological characteristics of the study population. Demographic characteristics included gender, gestational age, birthweight, 1 min and 5 min Apgar scores, type of delivery, maternal age, premature rupture of membranes, maternal chorioamnionitis, systemic antibiotics given to mother, maternal diabetes and preeclampsia. Clinicopathological features included respiratory support (including total oxygen absorption time during hospitalization, acceptance rate of invasive mechanical ventilation and non-invasive assisted ventilation), blood transfusion, hyperglycemia, supplementation of vitamin A (VA)and vitamin E (VE), bronchopulmonary dysplasia (BPD), NEC, intraventricular hemorrhage-III (IVH-III), intraventricular hemorrhage-IV (IVH-IV), periventricular leukomalacia (PVL) and LOS. BPD was diagnosed clinically (20). The diagnosis of NEC was based on clinical characteristics and imaging examinations (21). Intracranial lesions were determined by brain ultrasound or brain magnetic resonance imaging (22). LOS was depended on clinical manifestations and blood culture results (23).

### Outcome

The outcome of the study was the presence of ROP. With the improvement of the international classification of ROP, it was divided into stages 1–5 and some special lesions according to the severity of the disease: such as plus disease, pre-plus disease and aggressive posterior ROP. According to the severity of fundus lesions, this paper was divided into mild ROP (stage 1 or stage 2 disease in zone II and III without additional disease), severe ROP (stage ≥3 disease, zone I disease, pre-threshold disease, threshold disease, plus disease, aggressive posterior ROP) (24). ROP was diagnosed by ophthalmologist using the RetCam III digital camera (Clarity Medical systems, Inc., United States). Before ophthalmologic examinations, infants were deprived of food and water for 2 h. Infant pupils were dilated with mydriatic eyedrops (0.5% tropicamide, 3 times for 10 min per time), followed by application of ocular topical anesthetic based on these preparations, professional ophthalmologist put the camera lens on the surface of the cornea and took photos of fundus oculi.

### Statistical analysis

All statistical were analyzed using SPSS statistical software, version 21.0 (IBM Corp, Armonk, NY, United States). Normally distributed data were described by X ± S (mean ± standard deviation), and non-normally distributed data were described by median and interquartile range. Categorical variables were described with frequencies. The *χ*^2^ test was used to compare categorical variables while the T test and the nonparametric Mann-Whitney U rank sum test were used to compare continuous data. The association between probiotics and ROP was analyzed using univariate and multivariate logistic regression.

## Results

### Demographic characteristics of study population

A total of 443 preterm infants met the inclusion criteria, of which 264 were not supplemented with probiotics, 179 were supplemented with probiotics. There were 121 newborns with ROP (73 patients had stage 1, 24 had stage 2, 12 had stage 3, 2 had stage 4, 0 had stage 5, 8 had plus and 2 had aggressive posterior ROP), including 30 cases of severe ROP. There were statistically significant differences in gestational age, birthweight and one-minute Apgar score between with probiotic group and without probiotic group (*P* < 0.05). The demographic characteristics of the study population were detailed in Supplementary Table S1.

### Clinicopathological characteristics of the study subjects

The total oxygen inhalation time and the acceptance rate of invasive mechanical ventilation were significantly different in preterm infants with and without probiotics(*P* < 0.05). There were also differences in the prevalence of BPD, ROP and IVH-III, IVH-IV, PVL between two groups (*P* < 0.05). The clinicopathological characteristics of the study subjects were shown in Supplementary Table S2.

### Univariate and multivariate logistic regression analysis of the association between probiotics and ROP

With ROP as the dependent variable and probiotics as the only covariate, unadjusted univariate logistic regression indicated that the OR value of the probiotics' supplementation group was 0.383 (0.240∼0.611) (*P* < 0.01). Adjusted for prevalence of gestational age, birth weight, 1 min Apgar score, total oxygen inhalation time, invasive mechanical ventilation acceptance rate, exposure to blood transfusion, oral VA, oral VE, the prevalence of BPD and IVH-III, IVH-IV, PVL OR value was 0.575 for the use of probiotics (0.333∼0.994) (*P* < 0.05) (Supplementary Tables S3–4).

## Discussions

In our study, univariate logistic regression analysis showed that probiotics had a protective effect on ROP in preterm infants less than 32 weeks gestational age and birthweight <1500 g. The adjusted multivariate logistic regression results were consistent with the univariate logistic regression results, but the effect of probiotics was attenuated.

Probiotics are a class of active microorganisms that can colonize the gastrointestinal tract, and have gradually attracted researchers' attention as biological agents (25). Previous studies have found that appropriate probiotic supplementation can benefit the host by modulating local and systemic immunity (26). Studies from rodents suggest that gut dysbiosis may be associated with the development and exacerbation of many ophthalmic diseases such as diabetic retinopathy, age-related macular degeneration, choroidal neovascularization, uveitis and glaucoma. Gut-eye axis has become a new focus of basic and clinical research in ophthalmology (27). A recent study by the team of Professor Dimitra Skondra find that the microbiome profile of infants with severe ROP is significantly enriched in Enterobacteriaceae (16), therefore, the relationship between probiotics and retinopathy is worth expecting. Recently, a meta-analysis reports that the available evidence does not support any significant effect of probiotics on reducing the incidence of ROP (17). However, the authors also emphasize that their results should be interpreted with caution, as the included trials varied widely in terms of inclusion criteria (i.e., birthweight and gestational age), timing and dosage of probiotics application, and probiotics formulations. In addition, ROP is not the primary outcome in any of the trials, and the definition of severe ROP varies from study to study and no separate data is available for the population at highest risk of ROP (i.e., very preterm or very low birthweight infants). Therefore, the effect of probiotic supplementation on ROP in preterm infants is still unclear. Our study was first to use ROP as the primary outcome on which probiotics affect. We found that probiotics supplementation was associated with reduced incidence of ROP in preterm infants <32 weeks and birthweight <1500 g.

There may be several hypothesized mechanisms for the protective effect of probiotics on ROP. First, colonization of the gut microbiota often occurs during and after delivery. Various factors in early life influence the development and diversity of normal microbes, disrupting the balance between protective microbes and pathogenic microbes. These changes may be associated with the angiogenesis and the pathways related to ROP development. Therefore, probiotic supplementation probably can reduce the occurrence of ROP by regulating the composition of intestinal flora (28, 29). Second, probiotics may affect retinal vascular development in the first stage of ROP marked by insulin-like growth factor-1 deficiency, which plays a key role in vascular endothelial growth factor-driven angiogenesis (30, 31). Third, in addition, the performance of probiotics' antioxidant properties also helps to reduce the incidence of ROP (32, 33).

However, limitations of the present study should be noted. First, this study was a single-center retrospective study in which the study population was grouped according to probiotics use and parental refusal to use probiotics, while it can affect the result of this study. Second, although our study showed that the use of probiotics had a protective effect on ROP, it failed to analyze the differences among various types of probiotics, the dosages and the durations of continuous use. Third, retinopathy is a disease affected by multiple risk factors, therefore, factors not included may affect the results of this study. Fourth, the subjects included in this study were in-hospital cases, and there was no follow-up after discharge. Finally, different ophthalmologists might draw different diagnosis.

## Conclusion

In summary, probiotics were one of the factors affecting ROP in our study. After adjusting for covariates such as gestational age, birth weight, 1-minute Apgar score, total oxygen absorption time, invasive mechanical ventilation acceptance rate, blood transfusion, oral VA, oral VE, BPD, IVH-III, IVH-IV, PVL, we found that probiotics was related with a reduced risk of ROP in preterm infants with gestational age <32 weeks and birthweight <1500 g. Future studies enlarge sample capacity, extend to prospective studies and investigate the mechanism of how probiotics affect the ROP.

## Data Availability

The original contributions presented in the study are included in the article/**Supplementary Material**, further inquiries can be directed to the corresponding author.
