# Supplementary material for: The relationship between probiotics and retinopathy of prematurity in preterm infants: A population-based retrospective study in China
Source: Front Pediatr. 2023 Feb 21;11:1055992. doi: 10.3389/fped.2023.1055992 (PMC9989163; doi:10.3389/fped.2023.1055992)
Supplement: Supplementary file 1 [file Table1.docx]

Supplementary Material

Table 1. Demographic characteristics of the study population

|  | Without probiotics (N=264) | With probiotics (N=179) | χ^2^ /t | P |
| --- | --- | --- | --- | --- |
| **Gender** |  | | 0.022 | 0.882 |
| Male | 142（54%） | 95（53%） |  |  |
| Female | 122（46%） | 84（47%） |  |  |
| **Gestational(wks.)** | 29.1±1.6 | 29.7±1.3 | -4.124 | 0.000 |
| **Birth weight（g）** | 1138.3±213.1 | 1223.0±158.9 | -4.787 | 0.000 |
| **Apgar 1** |  | | 5.878 | 0.015 |
| ≥8 | 187（71%） | 145（81%） |  |  |
| ＜8 | 77（29%） | 34（19%） |  |  |
| **Apgar 5** |  | | 1.282 | 0.257 |
| ≥8 | 249（94%） | 173（97%） |  |  |
| ＜8 | 15（6%） | 6（3%） |  |  |
| **Type of delivery** |  | | 1.185 | 0.276 |
| Vagina | 151（57%） | 93（52%） |  |  |
| Cesarean | 113（43%） | 86（48%） |  |  |
| **Maternal age (year)** |  | | 1.538 | 0.215 |
| ≤30 | 150（57%） | 91（51%） |  |  |
| ＞30 | 114（43%） | 88（49%） |  |  |
| **Premature rupture of membranes** |  | | 0.277 | 0.598 |
| NO | 176（67%） | 115（64%） |  |  |
| Yes | 88（33%） | 64（56%） |  |  |
| **Maternal chorioamnionitis** |  | | 1.408 | 0.235 |
| NO | 247（94%） | 162（91%） |  |  |
| Yes | 17（6%） | 17（9%） |  |  |
| **Systemic antibiotics given to mother** |  | | 0.473 | 0.491 |
| NO | 162（61%） | 104（58%） |  |  |
| Yes | 102（39%） | 75（42%） |  |  |
| **Maternal diabetes** |  | | 1.438 | 0.231 |
| NO | 206（78%） | 148（83%） |  |  |
| Yes | 58（22%） | 31（17%） |  |  |
| **Maternal preeclampsia** |  | | 0.313 | 0.579 |
| NO | 198（75%） | 130（73%） |  |  |
| Yes | 66（25%） | 49（27%） |  |  |

Table 2. Clinicopathological characteristics of study subjects

|  | Without probiotics (N=264) | With probiotics (N=179) | χ^2^ /t/ Mann-Whitney U | P |
| --- | --- | --- | --- | --- |
| **Total oxygen absorption time（d）** | 26（27） | 17（21） | 17912.00 | 0.000 |
| **Invasive mechanical ventilation** |  | | 9.686 | 0.002 |
| NO | 165（63%） | 137（77%） |  |  |
| Yes | 99（37%） | 42（23%） |  |  |
| **Noninvasive mechanical ventilation** |  | | 0.193 | 0.660 |
| NO | 2（1%） | 3（2%） |  |  |
| Yes | 262（99%） | 176（98%） |  |  |
| **Blood transfusion** |  | | 2.776 | 0.096 |
| NO | 26（10%） | 27（15%） |  |  |
| Yes | 238（90%） | 152（85%） |  |  |
| **VA** |  | | 0.799 | 0.371 |
| NO | 214（81%） | 151（84%） |  |  |
| Yes | 50（19%） | 28（16%） |  |  |
| **VE** |  | | 0.297 | 0.586 |
| NO | 193（73%） | 135（75%） |  |  |
| Yes | 71（27%） | 44（25%） |  |  |
| **Hyperglycemia** |  | | 1.864 | 0.172 |
| NO | 148（56%） | 112（63%） |  |  |
| Yes | 116（44%） | 67（37%） |  |  |
| **ROP** |  | | 16.852 | 0.000 |
| NO | 173（66%） | 149（83%） |  |  |
| Yes | 91（34%） | 30（17%） |  |  |
| **BPD** |  | | 11.029 | 0.001 |
| NO | 159（60%） | 135（75%） |  |  |
| Yes | 105（40%） | 44（25%） |  |  |
| **NEC** |  | | 0.503 | 0.478 |
| NO | 219（83%） | 153（85%） |  |  |
| Yes | 45（17%） | 26（15%） |  |  |
| **IVH-III, IVH-IV, PVL** |  | | 3.968 | 0.046 |
| No | 229（87%） | 166（93%） |  |  |
| Yes | 35（13%） | 13（7%） |  |  |
| **LOS** |  | | 2.629 | 0.105 |
| NO | 160（61%） | 122（68%） |  |  |
| Yes | 104（39%） | 57（32%） |  |  |

Table 3：Risk factors of ROP in this study

|  | ROP | | | χ2 /t/ Mann-Whitney U | P |
| --- | --- | --- | --- | --- | --- |
|  | NO(N=322) | | Yes(N=121) |  |  |
| Gender |  | | | 0.073 | 0.787 |
| Male | 171（53%） | 66（55%） | |  |  |
| Female | 151（47%） | 55（45%） | |  |  |
| Gestational(wks.) | 29.6±1.4 | 28.5±1.6 | | 7.484 | 0.000 |
| Birth weight（g） | 1221.4±174.5 | 1042.4±196.2 | | 9.292 | 0.000 |
| Apgar 1 |  | | | 16.850 | 0.000 |
| ≥8 | 258（80%） | 74（61%） | |  |  |
| ＜8 | 64（20%） | 47（39%） | |  |  |
| Apgar 5 |  | | | 2.683 | 0.101 |
| ≥8 | 310（96%） | 112（93%） | |  |  |
| ＜8 | 12（4%） | 9（7%） | |  |  |
| Type of delivery |  | | | 0.255 | 0.614 |
| Vagina | 175（54%） | 69（57%） | |  |  |
| Cesarean | 147（46%） | 52（43%） | |  |  |
| Maternal age (year) |  | | | 0.031 | 0.860 |
| ≤30 | 176（55%） | 65（54%） | |  |  |
| ＞30 | 146（45%） | 56（46%） | |  |  |
| Premature rupture of membranes |  | | | 0.612 | 0.434 |
| NO | 215（67%） | 76（63%） | |  |  |
| Yes | 107（33%） | 45（37%） | |  |  |
| Maternal chorioamnionitis |  | | | 0.839 | 0.360 |
| NO | 295（92%） | 114（94%） | |  |  |
| Yes | 27（8%） | 7（6%） | |  |  |
| Systemic antibiotics given to mother |  | | | 0.334 | 0.563 |
| NO | 196（61%） | 70（58%） | |  |  |
| Yes | 126（39%） | 51（42%） | |  |  |
| Maternal diabetes |  | | | 0.007 | 0.934 |
| NO | 257（80%） | 97（80%） | |  |  |
| Yes | 65（20%） | 24（20%） | |  |  |
| Preeclampsia |  | | | 1.848 | 0.174 |
| NO | 244（76%） | 84（69%） | |  |  |
| Yes | 78（24%） | 37（31%） | |  |  |
| Total oxygen absorption time | 17（20） | 38（31） | | 8558.5 | 0.000 |
| Invasive mechanical ventilation |  | | | 51.954 | 0.000 |
| NO | 251（78%） | 51（42%） | |  |  |
| Yes | 71（22%） | 70（58%） | |  |  |
| Noninvasive mechanical ventilation |  | | | 0.136 | 0.712 |
| NO | 4（1%） | 1（1%） | |  |  |
| Yes | 318（99%） | 120（99%） | |  |  |
| Blood transfusion |  | | | 6.034 | 0.014 |
| NO | 46（14%） | 7（6%） | |  |  |
| Yes | 276（86%） | 114（94%） | |  |  |
| VA |  | | | 4.641 | 0.031 |
| NO | 273（85%） | 92（76%） | |  |  |
| Yes | 49（15%） | 29（24%） | |  |  |
| VE |  | | | 7.945 | 0.005 |
| NO | 250（78%） | 78（64%） | |  |  |
| Yes | 72（22%） | 43（36%） | |  |  |
| Hyperglycemia |  | | | 3.812 | 0.051 |
| NO | 198（61%） | 62（51%） | |  |  |
| Yes | 124（39%） | 59（49%） | |  |  |
| BPD |  | | | 67.129 | 0.000 |
| NO | 250（78%） | 44（36%） | |  |  |
| Yes | 72（22%） | 77（64%） | |  |  |
| NEC |  | | | 2.656 | 0.103 |
| NO | 276（86%） | 96（79%） | |  |  |
| Yes | 46（14%） | 25（21%） | |  |  |
| IVH-III, IVH-IV, PVL |  | | | 5.586 | 0.018 |
| No | 294（91%） | 101（83%） | |  |  |
| Yes | 28（9%） | 20（17%） | |  |  |
| LOS |  | | | 1.784 | 0.182 |
| NO | 211（66%） | 71（59%） | |  |  |
| Yes | 111（34%） | 50（41%） | |  |  |
| Probiotics |  | | | 16.852 | 0.000 |
| No | 173（54%） | 91（75%） | |  |  |
| Yes | 149（46%） | 30（25%） | |  |  |

Table 4：Univariate and multivariate logistic regression analyses for the association between probiotics and ROP

|  | β | P | OR (95% CI) |
| --- | --- | --- | --- |
| Probiotics (yes vs no) |  | | |
| Univariate analysis | -0.960 | 0.000 | 0.383（0.240~0.611） |
| Adjust model | -0.553 | 0.047 | 0.575（0.333~0.994） |
